# The psychosocial dimension of housing in Nunavik: does social support vary with household crowding?

**DOI:** 10.17269/s41997-022-00716-7

**Published:** 2022-12-19

**Authors:** Charles-Olivier Simard, Mylene Riva, Philippe Dufresne, Karine Perreault, Gina Muckle, Natalia Poliakova, Mireille Desrochers-Couture, Christopher Fletcher, Caroline Moisan, Sarah Fraser, Richard Bélanger, Yohann Courtemanche, Simona Bignami

**Affiliations:** 1 Département de démographie, Université de Montréal, Pavillon Lionel-Groulx, 3150 Jean Brillant St, Montreal, QC H3T 1N8 Canada; 2 Canada Research Chair in Housing, Community, and Health; Institute for Health and Social Policy, McGill University, Montreal, QC Canada; 3 Department of Geography, McGill University, Montreal, QC Canada; 4 École de Santé Publique, Université de Montréal, Montreal, QC Canada; 5 École de psychologie, Université Laval, Québec, QC Canada; 6 Centre de recherche du CHU de Québec, Université Laval, Québec, QC Canada; 7 Département de médecine sociale et préventive, Université Laval, Québec, QC Canada; 8 Faculté des arts et des sciences - École de psychoéducation, Université de Montréal, Montreal, QC Canada

**Keywords:** Inuit, Nunavik, Social support, Overcrowding, Housing, Household, Inuit, Nunavik, soutien social, surpeuplement, logement, ménage

## Abstract

**Objectives:**

Studies show that living in overcrowded households can contribute to the erosion of social support, which is an important factor in health and well-being. In this study, we examine the relationship between household crowding and social support for Inuit living in Nunavik (hereafter referred to as Nunavimmiut), a region where housing shortages are considered a serious public health problem. We assess whether overcrowding is associated with lower levels of perceived social support and whether this association varies by gender and age group.

**Methods:**

Cross-sectional data are from *Qanuilirpitaa?* the 2017 Nunavik Health Survey (*N* = 1306; aged 16 years and older). A perceived social support index was derived from answers to questions related to three different components of social support: positive interaction, emotional support, and love and affection. Associations between overcrowding (more than one person per room) and perceived social support were assessed using weighted linear and logistic regressions, adjusted for several factors. Sex- and age-stratified analyses were also conducted.

**Results:**

Nunavimmiut report significantly lower levels of social support when living in overcrowded households, independently of other covariates. Analyses stratified by sex and age further show that the detrimental association between overcrowding and perceived social support is higher and stronger for men and older adults (both men and women 55 years and older).

**Conclusion:**

Overcrowding is associated with lower levels of perceived social support, which is a key component of health for the general population and for Nunavimmiut. Future research should examine the factors creating stronger associations between overcrowding and lower social support for men and older adults.

## Résumé

**Objectifs:**

Des études montrent que le fait de vivre dans un ménage surpeuplé peut contribuer à l’érosion du soutien social, qui est un facteur important de santé et de bien-être. Dans cette étude, nous examinons la relation entre le surpeuplement des ménages et le soutien social chez les Inuits du Nunavik, une région où la pénurie de logements est considérée comme un grave problème de santé publique. Nous évaluons si le surpeuplement est associé à des niveaux plus faibles de soutien social perçu chez les Inuits vivant au Nunavik (ci-après appelés Nunavimmiut), et si cette association varie selon le sexe et le groupe d’âge.

**Méthodes:**

Les données transversales proviennent de *Qanuilirpitaa?* l’enquête sur la santé au Nunavik de 2017 (N = 1 306; âgés de 16 ans et plus). Un indice de soutien social perçu a été dérivé des réponses aux questions relatives à trois composantes différentes du soutien social : interaction positive, soutien émotionnel, et amour et affection. Les associations entre le surpeuplement résidentiel (plus d’une personne par pièce) et le soutien social perçu ont été évaluées à l’aide de régressions linéaires et logistiques pondérées, ajustées pour plusieurs facteurs. Des analyses stratifiées par sexe et par âge ont également été réalisées.

**Résultats:**

Les Nunavimmiut déclarent des niveaux de soutien social significativement plus faibles lorsqu’ils vivent dans des ménages surpeuplés, indépendamment d’autres covariables. Les analyses stratifiées par sexe et par âge montrent en outre que l’association néfaste entre le surpeuplement et le soutien social perçu est plus élevée et plus forte chez les hommes et les adultes plus âgés (hommes et femmes de 55 ans et plus).

**Conclusion:**

Le surpeuplement résidentiel est associé à des niveaux inférieurs de soutien social perçu, lequel est une composante clé de la santé pour la population générale et pour les Nunavimmiut. Les recherches futures devraient examiner les facteurs en cause derrière l’association entre le surpeuplement résidentiel et l’affaiblissement du soutien social chez les hommes et les adultes plus âgés.

## Introduction

Social relationships are critical to individual and community well-being for Inuit (Fletcher et al., 2022; Richmond, 2009). International studies and research carried out with Inuit have highlighted the negative impacts of overcrowding on mental health (Evans et al., 2003; Gray et al., 2016; Riva et al., 2014a) with the weakening of social support posited as one of the mechanisms explaining this association (Evans & Lepore, 1993; Wells & Harris, 2007; Riva et al., 2014b). The objective of this study is to examine the association between overcrowding and perceived social support for Inuit living in Nunavik (hereafter referred to as Nunavimmiut), a region where more than half of the population lives in overcrowded households (Statistics Canada, 2017). The study also assesses whether associations between overcrowding and perceived social support vary across sex and age groups.

## Background

The Inuit homeland in the Canadian Arctic is an immense territory, stretching from northern Labrador to the far west of the Northwest Territories, passing through the central Canadian Arctic. Today, this vast territory constitutes Inuit Nunangat (“where the Inuit live” in Inuktitut), representing nearly 30% of Canada’s land mass. Nunavik, the region covered by our study, is one of the four political and administrative regions established within the limits of this traditional territory. It is made up of 14 remote villages and has a total population of 14,000 people, more than 90% of whom are Inuit.

Inuit occupation of the Arctic was accomplished by highly mobile groups of people with sophisticated knowledge and hunting technologies that focused on marine resources and migratory ungulates. This pattern of occupation, which favoured temporary, seasonal and cyclic habitation and household configurations, lasted until the middle of the twentieth century. In the context of the dawn of the Cold War and threats to the Canadian sovereignty in the Arctic, and the collapse of the fur market, and amid an acute socio-sanitary crisis caused by tuberculosis epidemics, the federal government made the decision to forcibly relocate Inuit into permanent communities (Bonesteel & Anderson, 2008; Duhaime, 1983). The construction of fixed dwellings became the crucial tenet of the settlement process (Duhaime, 1983) and was used as a tool for cultural assimilation, as Inuit were forced to enter the wage economy and adopt a sedentary lifestyle. Even though Inuit are actively advocating for new policies to support self-determination (Fabbi et al., 2017), an analysis of the power relations in the housing sector indicates that Inuit remain the executants of policies developed by non-Inuit and that their involvement in decisions regarding the nature and the distribution of resources is minimal (Therrien & Duhaime, 2017). Moreover, the increased reliance on wage earnings and the various housing practices that have been implemented over the last decades (e.g. housing allocation rules and rent scales) have created economic disparities among Inuit and, in doing so, have disrupted sharing practices that are highly integrated in kin-oriented social support networks (Stern, 2005; Wenzel, 2000). Housing is an important determinant of Inuit health because of its impacts on family relationships and cultural identity, which are integral parts of Inuit sense of home and Inuit conceptualizations of health (Christensen, 2016; Fletcher et al., 2021; Perreault et al., 2020).

Nowadays, in Nunavik, nearly 90% of the population resides in social housing owned and managed by the Kativik Municipal Housing Bureau (KMHB) (Société d’habitation du Québec, 2014). The allocation of housing in the region is decided by a point system that determines the “imperative” nature of applicants’ housing needs (Société d’habitation du Québec, 2014). In such a system, the rent scale is adapted to the size of the dwelling and to household income, with more vulnerable or at-risk families given priority for new units (Société d’habitation du Québec, 2014). Each village in Nunavik has a waitlist for social housing units, although it can take years for applicants to be allocated a new dwelling.

The results of the 2015 *Nunavik Housing Needs Survey* suggested that, in addition to the 2884 existing public housing units, more than 1000 new dwellings should be built in order to meet the housing needs of Nunavimmiut (Deloitte LLP and its affiliates, 2019, p. 14; Standing Senate Committee on Aboriginal Peoples, 2017). The 2016 Canadian census indicated that nearly 52% of the population in Nunavik lived in housing that would be considered overcrowded (Statistics Canada, 2017). The severe housing shortage and resulting overcrowding have been described and decried by multiple public reports, most of which have highlighted that the lack of housing in Nunavik (and more generally across Inuit Nunangat) has been a chronic problem since the very beginning of forced settlement (Knotsch & Kinnon, 2011). Inuit organizations have called for additional public funds—over two billion dollars across Inuit Nunangat (Standing Senate Committee on Aboriginal Peoples, 2017)—to address this major issue (Inuit Tapiriit Kanatami, 2016).

The negative consequences of overcrowding^1^ on mental health have been demonstrated by several studies carried out among Inuit in the last decade. In a cross-sectional study, Riva et al. (2014a, 2014b) showed that allostatic load—a biological marker of chronic stress—was higher for Nunavimmiut living in overcrowded households (defined as households with more than one person per room), independently of socio-demographic and economic covariates. While women’s allostatic load was especially associated with overcrowding, analyses stratified by sex did not reveal significant association with overcrowding for men. One explanation provided by the authors was that women play a very prominent role in the organization of domestic activities in traditional Inuit culture, so the loss of control resulting from living in overcrowded houses would affect a rather crucial component of their daily life. This interpretation is supported by studies showing that a lower sense of control can amplify the detrimental effects of a stressful situation on a person’s health and well-being (Elliot et al., 2018; Lachman & Weaver, 1998). More recently, a study conducted in Nunavik and Nunavut reported that overcrowding was associated with a lower “sense of home”, a measure reflecting participants’ perception of their home in relation to various aspects, such as space, identity, control, privacy, and family relationships (Perreault et al., 2020). Among Inuit adults who were rehoused to a new dwelling, psychological distress and perceived stress were significantly lower 15 to 18 months after the move (Riva et al., 2020). The main predictors of psychological distress improvement were the reduction in the number of adults per household and the increase in sense of home (Perreault et al., 2022a). However, two recent longitudinal studies did not observe a significant association between overcrowding measured at baseline and psychological health measured at follow-up for Inuit adolescents in Nunavik (Pepin et al., 2018) and Greenlandic adults (Hansen et al., 2020). Several factors, including different study designs, time to follow-up, age groups, and attrition bias, could explain these divergent results.

Research suggests that the weakening of social support is one key mechanism through which overcrowding affects mental health (Evans & Lepore, 1993; Wells & Harris, 2007; Riva et al., 2014a, 2014b). The hypothesis is that overcrowding generates frequent unwanted interactions and tensions among household members, which, in turn, leads to self-isolation^2^. Social withdrawal would therefore be an adaptative behavioural response to unwanted social interactions and the accompanying loss of control that one may perceive when exposed to these conditions. But when adopted in the long run, this coping strategy could lead to the “disruption of socially supportive relationships” (Evans & Lepore, 1993: p. 1827). Weakening social support, ultimately, is a risk factor for psychological distress.

This may be particularly relevant for Inuit living in overcrowded homes, given the importance of social support within Inuit’s conceptualization of health (Fletcher et al., 2022). Indeed, a recently developed model of Nunavik Inuit Health identified three components of health: *Ilusirsusiarniq*, which closely relates to “bodily health” and conveys a condition of normalcy; *Qanuinngisiarniq*, which is a broadly defined sense of well-being that encompasses feelings of being unworried, without pain, comfortable, free of emotional distress, and happy; and *Inuuqatigiitsianiq*, a third component that is imbued with the quality of social relationships (Fletcher et al., 2022). The latter refers to an ideal form of social well-being that is manifest through relatedness, caring, and quality of interpersonal relations among people who share the same place—such as a house. Good relationships are viewed by Nunavimmiut as essential to health and well-being, so fostering Inuuqatigiitsianiq in families is vital (Fletcher et al., 2022). In contrast, poor relations can trigger and accentuate tensions between people. As shown by this model of health in Nunavik, social relationships are intrinsically, and indissociably, related to health and well-being for Inuit.

In a similar vein, studies have shown the importance of social support as a protective factor against psychological distress and other important social and health issues in Inuit communities, such as drug use (Cao et al., 2018) and suicide (Kral, 2016). In a study conducted with Greenland Inuit (Riva et al., 2014a, 2014b), the association between overcrowding and poor mental well-being was partly explained through reduced social support, independently of other socio-demographic and economic characteristics. This mediation was significant for women but not for men. More recently, a qualitative study conducted in Nunavut showed that living in overcrowded conditions implies constant negotiations for limited space, food, and water, conditions in which people feel they lack control and that instil a sense of “powerlessness” (Perreault et al., 2022b). The study suggests that this ultimately leads to social tensions that undermine supportive family relationships and mental well-being, a finding consistent with the theoretical model originally proposed by Evans and Lepore (1993).

In addition, the association between overcrowding and the weakening of social support may also vary by age. In a critique of the use of objective overcrowding measures for research and policy in the Arctic, Lauster and Tester (2010) suggested that the housing shortage in Inuit Nunangat primarily affects the younger generations. Overcrowding could therefore be more strongly associated with lower social support for younger age groups, although the authors did not consider the potential mediating role of social support when discussing the consequences of the housing crisis on the younger generations.

Given the existing literature, we would therefore expect overcrowding to be associated with lower levels of social support for Nunavimmiut, particularly for women and younger age groups.

## Data and methods

Cross-sectional data from *Qanuilirpitaa?* the 2017 Nunavik Health Survey was used. Qanuilirpitaa is a joint initiative between the Nunavik Regional Board of Health and Social Services, the Institut national de santé publique du Québec, and academic researchers. Several regional organizations were also involved in the survey. Data were collected onboard the research ship Amundsen which sailed to all 14 communities in Nunavik in October 2017; Nunavimmiut aged 16 years and older were invited to participate. Data were collected from computerized and interviewer-administered questionnaires (available in Inuktitut, English, and French), clinical and laboratory testing, and medical records review. The Qanuilirpitaa? Survey was approved by the Research Ethics Board of the Centre hospitalier universitaire de Québec-Université Laval. Overall, 1326 Nunavimmiut participated in *Qanuilirpitaa?*, nearly 18% of the 7560 Inuit aged 16 years and older who were included in the 2016 census. Detailed information on survey procedures is provided elsewhere (Hamel et al., 2020).

### Measures

#### Perceived social support

Perceived social support was assessed using five questions adapted from the Aboriginal Peoples Survey (Statistics Canada, 2001) addressing positive interaction (question 1), emotional support (questions 2 to 4), and love and affection (question 5). These questions included the following: (1) How often do you find that you have someone to have a good time with? (2) How often do you have someone to talk to if you feel troubled or for some reason need emotional support? (3) How often do you have someone you can count on when you need advice? (4) How often do you have someone you can count on to listen to you when you need to talk? (5) How often do you have someone who shows you love and affection? Possible answers included: all of the time, most of the time, sometimes, rarely, and never. These responses were scored from 5 to 1, respectively. Scores for each question were then added to form a continuous social support index ranging from 5 (the lowest score) to 25 (the highest score) (Cronbach *α* = 0.79).

To examine possible differences in associations between overcrowding and the above-mentioned components of social support, the latter were modelled as three dichotomous variables. For the question relating to positive interaction and to love and affection components, responses “all of the time” or “most of the time” were grouped and compared with the other response categories. For emotional support, participants answering “all of the time” or “most of the time” to the three variables of the component were grouped and contrasted to participants answering otherwise.

#### Household overcrowding

Household overcrowding was measured using a dichotomous variable generated from the number of people per room (PPR), with 0 indicating a non-crowded household (less than one person per room) and 1 indicating an overcrowded household (more than one person per room). Frequently used to measure overcrowding in the Arctic (see notably Hamel et al., 2020; Minich et al., 2011; Riva et al., 2014a, 2014b), the PPR ratio was computed using information on the number of rooms in the dwelling that was provided by KMHB and data collected during the survey process on the number of people living in the house of participants. The number of rooms includes the kitchen, living room, and dining room, but excludes the bathroom, hallways, and closets.

#### Covariates

Age group (16–30 years; 31–54 years; 55 years and older), sex, personal income, and conjugal status were considered as sociodemographic and economic covariates (see Table 1 for variables’ categorization). These are fundamental control variables included in most psychosocial studies. For example, a study reported that older age groups (aged 55 years and older), single adults, and men, specifically, report lower levels of social support among Canadian Inuit (Richmond, 2009). Additionally, given that there are several disparities in socioeconomic (Riva et al., 2020), housing (Riva et al., 2020a), social support (Muckle et al., 2020a), and health (Muckle et al., 2020b) conditions between the villages located on the Hudson and Ungava coasts of Nunavik, administrative region was included as a geographical covariate.

**Table 1:** Sociodemographic and household characteristics of the 1326 respondents to the Q2017 survey

	Total % (95% CI)	Overcrowding (8.7% missing)	
		≤ 1 ppr % (95% CI)	> 1 ppr % (95% CI)
Sex			
Women	50.4 (NA^a^)	49.2 (46.8, 51.5)	53.0 (49.2, 50.3)
Men	49.6 (NA^a^)	50.9 (48.5, 53.2)	47.0 (42.5, 51.6)
Age (years)			
16–30	43.9 (NA^a^)	40.2 (37.7, 42.7)	50.9 (46.0, 55.8)^*^
31–54	39.3 (37.9, 40.9)	39.6 (36.5, 42.7)	40.9 (36.2, 45.9) ^*^
≥ 55	16.8 (15.4, 18.4)	20.3 (18.0, 22.8)	8.2 (5.8, 11.5) ^*b^
Relationship status			
In a relationship	52.5 (49.5, 55.4)	49.8 (46.0, 53.5)	59.7 (54.2, 64.9) ^*^
Single	47.5 (44.6, 50.5)	50.2 (46.5, 54.0)	40.3 (35.1, 45.8) ^*^
Region			
Hudson coast	56.7 (56.1, 57.3)	57.0 (54.5, 59.5)	62.6 (57.9, 67.0)
Ungava coast	43.3 (42.8, 43.9)	43.0 (40.5, 45.4)	37.4 (33.0, 42.1)
Personal annual income			
< $20,000	46.1 (43.2, 49.0)	44.4 (40.6, 48.4)	51.7 (46.5, 56.9)
$20,000–$39,999	17.6 (15.4, 20.1)	20.2 (17.3, 23.4)	14.4 (10.9, 18.8)
$40,000–$59,999	11.3 (9.4, 13.4)	11.6 (9.3, 14.4)	11.1 (7.9, 15.3)^b^
≥ $60,000	11.8 (10.1, 13.7)	13.0 (10.8, 15.6)	7.7 (5.0, 11.5)^b^
Missing	13.2 (11.5, 15.3)	10.8 (8.8, 13.1)	15.1 (11.6, 19.6)
Overcrowding			
> 1 person per room	32.8 (29.9, 35.8)		
≤ 1 person per room	67.2 (70.1, 64.2)		
Social support score—mean (95% CI)	18.3 (18.1, 18.6)	18.5 (18.2, 18.7)	18.0 (17.6, 18.5)
Positive interactions^c^	67.7 (64.6, 70.7)	67.8 (64.0, 71.4)	66.0 (60.1, 71.4)
Love and affection^c^	73.2 (70.4, 75.8)	76.1 (72.9, 78.9)	68.5 (63.1, 73.4)^*^
Emotional support^c^	30.3 (27.6, 33.2)	32.0 (28.4, 35.8)	26.3 (22.0, 31.1)^†^

^*^Wald test—distribution of variables statistically different across categories of overcrowding at *p* < 0.05

^†^Wald test—distribution of variables statistically different across categories of overcrowding at *p* < 0.10

^a^Non-available. Categories of variables used to create weights. The estimate shows no variability; confidence intervals are the same as the estimate

^b^Coefficient of variation > 15

^c^Participants answering “all of the time” and “most of the time”

### Statistical analysis

Associations between dependent and independent variables were measured using linear and logistic regression models, adjusting for covariates. Because previous research suggests that overcrowding effects on mental health and well-being may vary by age and sex (Riva et al., 2014a, 2014b; Lauster & Tester, 2010), we also conducted analyses that stratified sex and age groups (age 16–29, 30–54, and 55 and older). Analyses were weighted to be representative of the population of Nunavik aged 16 years and older. Ninety-five percent confidence intervals were calculated using bootstrapped variance estimates, and coefficients of variation (CV) were computed. When the percentage of missing data was above 10%, they were computed as a distinct category of the variable.

### Integrating the Inuit perspective into research

As with all research produced with *Qanuilirpitaa?* data, this study underwent a review process by a committee comprised of Nunavimmiut and representatives of Nunavik organizations. We first shared the manuscript with members of this committee and then presented the research in a virtual meeting. Committee members then shared their comments and suggested ways to interpret the results. These comments have been integrated into the present version of the article submitted for publication. The comments improved the article and ensured its relevance from a Nunavik perspective.

## Results

Characteristics of the population and comparisons according to overcrowding status are presented in Table 1. The sample comprised approximately equal numbers of men (49.6%) and women (50.4%). Fifty-two percent of Nunavimmiut were in a relationship, while 48% were single. Close to half (46.1%) of the participants reported an annual income below $20,000. About one third of Nunavimmiut aged 16 years and older were living in an overcrowded household (more than 1 person per room). More women (53%) than men (47%) lived in overcrowded dwellings, but the difference was not statistically significant. The mean score of the social support index was marginally lower (*p* < 0.10) at 18.0 for people living in overcrowded households, compared to a score of 18.5 for those living in non-crowded households (*p* < 0.10). The 16–30 age group, which represented 43.9% of the population, represented 50.9% of those living in overcrowded households. Conversely, while representing 16.8% of the population, the 55 and over age group only constitutes 8.2% of those living in overcrowded households.

### Household overcrowding and social support

Table 2 presents results for the associations between household overcrowding and perceived social support, adjusted for participants’ covariates. Household overcrowding was significantly and negatively associated with social support. More specifically, compared with people not living in overcrowded households, those living in a house with more than one person per room were significantly more likely to have a lower score (*β*: − 0.67; 95% CI: − 1.18, − 0.16) on the social support index, independently of other covariates. For the different components of social support, the odds of receiving love and affection were almost half (OR: 0.54; 95% CI: 0.40, 0.74) for participants living in overcrowded households compared with those for participants living in non-crowded households. Overcrowding was also negatively and significantly associated with emotional support (AOR: 0.72; 95% CI: 0.53–0.97) but not with positive interactions.

**Table 2:** Social support index, its subcomponents and overcrowding in Nunavik 2017, adjusted for covariates, Q2017 survey

	Social support *β* (95% CI)	Subcomponents of social support (odds ratio (95% CI))		
		Positive interactions	Emotional support	Love and affection
Overcrowding (> 1 person per room)	− 0.67 (− 1.18, − 0.16)*	0.84 (0.62, 1.16)	0.72 (0.53, 0.97)*	0.54 (0.40, 0.74)*
Covariates				
Age (years)				
16–30 (REF)				
31–54	− 0.14 (− 0.68, 0.41)	0.64 (0.45, 0.91)*	1.02 (0.73, 1.43)	0.87 (0.60, 1.27)
≥ 55	− 1.09 (− 1.71, − 0.46)*	0.43 (0.29, 0.65)*	0.77 (0.53, 1.12)	0.61 (0.39, 0.94)*
Sex				
Women (REF)				
Men	− 1.07 (− 1.55, − 0.58)*	0.78 (0.58, 1.05)	0.53 (0.39, 0.72)*	0.73 (0.53, 1.01)
Relationship status				
In a relationship	0.95 (0.49, 1.41)	0.99 (0.73, 1.35)	1.41 (1.07, 1.87)*	3.39 (2.43, 4.73)*
Income				
< $20,000 (Ref)				
$20,000–$39,999	0.05 (− 0.52, 0.62)	1.49 (0.99, 2.22)	0.92 (0.62, 1.36)	1.04 (0.69, 1.56)
≥$40,000	0.71 (0.05, 1.38)*	1.14 (0.77, 1.68)	2.06 (1.42, 2.97)*	1.55 (0.98, 2.44)
Missing	0.05 (− 0.73, 0.84)	1.15 (0.72, 1.82)	1.20 (0.77, 1.86)	1.27 (0.79, 2.03)
Region				
Hudson (REF)				
Ungava	− 0.22 (− 0.71, 0.27)	0.97 (0.75, 1.27)	1.03 (0.76, 1.38)	0.89 (0.65, 1.22)

*p < 0.05

### Stratification by sex and by age groups

To examine whether the association between overcrowding and perception of social support varied across sex and age groups, we conducted stratified analyses for each subcategory (Table 3). While the association between overcrowding and perceived social support was no longer significant for women, it remained significant for men (*β*: − 0.90, 95% CI − 1.77, − 0.04). The negative and significant association between overcrowding and social support also appeared to be stronger in the 55 years and older age group (*β*: − 1.58, 95% CI − 3.11, − 0.05) but not in younger age groups (age 16–29 and 30–54).

**Table 3:** Social support, its components and overcrowding^a^ stratified by sex or age groups, Q2017 survey

	Sex^1^		Age groups^2^		
	Men	Women	16–30 years old	31–54 years old	≥ 55 years old
Dependent variables					
Social support * β* (95% CI)	− 0.90 (− 1.77, − 0.04)*	− 0.44 (− 1.03, 0.14)	− 0.72 (− 1.46, 0.02)	− 0.35 (− 1.14, 0.44)	− 1.58 (− 3.11, − 0.05)*
Positive interactions Odds ratio (95% CI)	0.87 (0.50, 1.51)	0.83 (0.58, 1.18)	0.67 (0.43, 1.06)	0.83 (0.48, 1.42)	2.17 (0.74, 6.34)^b^
Love and affection Odds ratio (95% CI)	0.40 (0.24, 0.69)*	0.74 (0.51, 1.09)	0.45 (0.29, 0.70)	0.73 (0.42, 1.28)	0.42 (0.15, 1.16)
Emotional support Odds ratio (95% CI)	0.61 (0.33, 1.14)	0.81 (0.58, 1.00)	0.83 (0.52, 1.31)	0.74 (0.45, 1.21)	0.22 (0.07, 0.68)^c^*

^1^All models adjusted for age, relationship status, income, and region

^2^All models adjusted for sex, relationship status, income, and region

^a^ ≥ 1 ppr (person per room)  coefficient or odds ratio is presented

^b^One or more parameters could not be estimated in 1 out of 500 bootstrap replicates; standard error estimates include only complete replications

^c^One or more parameters could not be estimated in 2 out of 500 bootstrap replicates; standard error estimates include only complete replications

*p < 0.05

## Discussion

The objective of this paper was to examine whether overcrowding is associated with lower perception levels of social support for Nunavimmiut, independently of other sociodemographic and economic characteristics. The study also aimed to assess whether this association varies across age groups and sex.

As hypothesized, people living in overcrowded households reported lower levels of social support, independently of sociodemographic and economic covariates. Given that social support and close social relationships are key dimensions of health for Inuit (Baron et al., 2020; Fletcher et al., 2022), this finding can be considered culturally relevant for Nunavimmiut. It is also consistent with previous research showing that the disruption of socially supportive relations affects associations between overcrowding and mental health (Evans & Lepore, 1993; Wells & Harris, 2007), although we did not consider impacts on mental health in this paper.

Findings indicate that associations between overcrowding and perception of social support vary across sex and age groups. Contrary to results from a previous study conducted in Greenland (Riva et al., 2014a, 2014b), overcrowding for Nunavimmiut is negatively associated with social support for men, but not for women. One explanation for this finding may be the unobserved differences in household composition between men and women. The proportion of single parents is high among Inuit, with women most often taking care of children at home. It is possible that, when living in overcrowded households, women tend to live with their own children, whereas men disproportionately live with other adults^3^. The level and type of social support one receives may differ in these two situations. As data from *Qanuilirpitaa?* do not allow to determine the type of relationships between household members, we could not consider household composition in our analysis.

A study on the social determinants of Inuit health in Canada reported that Inuit elders (aged 55 years and older) were more likely than younger age groups to report lower levels of social support (Richmond, 2009). Our research adds to this finding by showing that, in association with overcrowding, perceived social support for Nunavimmiut aged 55 years and older was significantly lower, independently of other covariates. This was not the case for other age groups. Once again, unobserved differences in household composition between age groups could partly explain these age-stratified associations. Indeed, multi-generational living arrangements are known to be more prevalent for Inuit than for First Nations and Métis in Canada (Battams, 2019). Although multigenerational households are not necessarily overcrowded and may constitute a preferred type of living arrangement for some (Hervé & Laneuville, 2017), this may not be the case for everyone. Some elders can provide care and shelter to their children and grandchildren which, in some circumstances, can create financial and psychological stress (Sigouin et al., 2010). Others may live in their children’s or grandchildren’s house because of health issues and/or economical constraints. Both situations are likely to influence the perception of social support for older Nunavimmiut.

While discussing the results of this study with representatives from regional organizations in Nunavik, it was pointed out that many elders temporarily house their adult grandchildren who do not have access to housing. Communities suggested that this may be especially true for young men who have been incarcerated and are returning to their communities with no other place to stay. Confronted with some young men's problems, such as substance use and violent behaviour, many older adults can experience significant psychological distress. One Inuit representative suggested that this reality may explain the negative associations between overcrowding and perceived social support for elders and men highlighted in our study.

In any case, although elders represent only a small fraction of Nunavimmiut living in overcrowded households (close to 8% in our study), our results show that their perceived social support is lower than that for other age groups when living in such conditions, independently of other covariates. This leads us to think that Inuit elders’ specific housing needs and vulnerabilities may currently be misunderstood and underestimated. As this age group will represent a growing proportion of the Inuit population in the next decades (Statistics Canada, 2015), more research will be needed to address this knowledge gap.

### Limitations

The cross-sectional design of the Q2017 survey precludes analyzing the timing of events. Therefore, self-selection and reverse causality cannot be ruled out, as people with pre-existing lower levels of perceived social support may have ended up living in overcrowded households for reasons that were not considered in this paper. Cohort studies are needed to overcome this limitation. In another vein, the PPR ratio has been criticized for its lack of cultural relevance (Lauster & Tester, 2010). However, a recent study conducted with Nunavut and Nunavik Inuit showed that this “objective” overcrowding measure was strongly correlated with how Inuit perceived overcrowding (the “subjective” definition of overcrowding) (Perreault et al., 2020), which indicates that both measure the same concept. Another limitation concerns the measure of social support. Given the self-reported nature of the survey data, it is impossible to determine whether results point to differences in social support *in itself* or rather differences in the *perception* of social support. The sex-stratified associations highlighted in the previous section, for instance, may reflect variations in the perception of social support rather than actual differences in social support*.* Finally, we must also mention that the percentage of the population living in overcrowded households in our weighted sample (32.8%) is considerably lower than that reported in the 2016 Canadian Census (52%) for the Nunavik region. We therefore suspect that Nunavimmiut living in the most severely overcrowded households constitute a hard-to-reach group underrepresented in our sample. From this perspective, our results could underestimate the negative association between overcrowding and social support for Nunavimmiut.

## Conclusion

The chronic lack of adequate housing has influenced the health and well-being of Inuit since the nascence of today’s Arctic communities. The pathways through which housing influences health are complex and modulated by many social and economic factors. In this study, we have examined how household overcrowding is associated with social support, a key component of health and well-being for Nunavimmiut (Fletcher et al., 2022). Findings further indicate gender- and age-specific patterns regarding this association which could be the focus of targeted public health interventions and suggests direction for further research. Qualitative and intervention studies are needed to deepen the understanding on this issue and determine whether the cross-sectional associations highlighted in this paper are causal. In particular, exploring the social and cultural dynamics by which overcrowding may affect the psychosocial dimension of housing, including sex- and age-specific experiences of socially supportive relationships, could bring valuable knowledge to the field. Of course, these scientific considerations do not mitigate the fact that the lack of affordable housing is an urgent issue that exacerbates social inequalities between Inuit communities and the general Canadian population. Inuit Tapiriit Kanatami, which represents Inuit interests nationally, advocates for direct and sustainable federal funding and recognition of the role of Inuit organizations in all aspects of housing in order to improve housing access in Inuit communities (Inuit Tapiriit Kanatami, 2019). Governments do not have to wait for scientific research to understand the full causality between housing and health to respond quickly and affirmatively to this request.

## Contributions to knowledge

What does this study add to existing knowledge?

- This study shows for the first time that living in an overcrowded household is associated with decreased social support among Nunavimmiut (Inuit of the Nunavik region), particularly among men and those aged 65 years and older, independently of confounding variables such as marital status, income, and subregion of residence. This finding is important both because overcrowded households are considered a major public health problem in Nunavik and because social support is a recognized protective factor against psychological distress that is central to the Inuit conceptualization of health.

What are the key implications for public health interventions, practice or policy?

- Our study adds to existing research that highlights the importance of improving access to housing among Nunavimmiut in order to promote health and well-being in the region.

## Data Availability

Inquiries regarding access to the data should be addressed to the survey’s *Data Management Committee*.

## Footnotes

1 Definitions of overcrowding are generally based on two common elements, namely the ratio between the number of persons and the number of rooms (or bedrooms) in the dwelling, and a threshold beyond which the ratio indicates that the household is overcrowded. Characteristics such as gender, age, and type of family relationship may be considered additionally, as in the National Occupancy Standard (NOS) definition used in the Canadian Census.

2 The isolation we are discussing here does not have to be *spatialized*. Indeed, whereas people living in overcrowded households lack the necessary space to temporarily withdraw from unwanted interactions, they can simply withdraw from social interactions by showing less concern for their relatives (see Evans & Lepore, 1993, for instance).

3 This hypothesis seems to be coherent with the Q2017 Housing and Drinking Water report showing that a higher proportion of men than women live in adult-only households (Riva et al., 2020a, 2020b)

## References

[CR1] Baron, M., Fletcher, C., & Riva, M. (2020). Aging, health and place from the perspective of elders in an Inuit community. *Journal of Cross-Cultural Gerontology*, 1–21. 10.1007/s10823-020-09398-5 32409899

[CR2] Battams, N. (2019). *Sharing a roof: Multi-generational homes in Canada (2016 Census Update).* (Transitions). The Vanier Institute of The Family. https://vanierinstitute.ca/multigenerational-homes-canada/

[CR3] Bonesteel, S., & Anderson, E. (2008). *Les relations du Canada avec les Inuit: Histoire de l’élaboration des politiques et des programmes*. Affaires indiennes et du Nord Canada.

[CR4] Cao L, Burton VS, Liu L (2018). Correlates of illicit drug use among Indigenous peoples in Canada: A test of social support theory. International Journal of Offender Therapy and Comparative Criminology.

[CR5] Christensen J (2016). Indigenous housing and health in the Canadian North: Revisiting cultural safety. Health & Place.

[CR6] Deloitte LLP and its affiliates. (2019). *Social housing needs assessment for the 14 Inuit communities of Nunavik* (p. 37). https://www.nunivaat.org/doc/publication/Deloitte-01.pdf

[CR7] Duhaime, G. (1983). La sédentarisation au Nouveau-Québec inuit. *Etudes/Inuit/Studies*, 25–52.

[CR8] Elliot AJ, Turiano NA, Infurna FJ, Lachman ME, Chapman BP (2018). Lifetime trauma, perceived control, and all-cause mortality: Results from the Midlife in the United States Study. Health Psychology.

[CR9] Evans GW, Lepore SJ (1993). Household crowding and social support: A quasiexperimental analysis. Journal of Personality and Social Psychology.

[CR10] Evans GW, Wells NM, Moch A (2003). Housing and mental health: A review of the evidence and a methodological and conceptual critique. Journal of Social Issues.

[CR11] Fabbi NC, Rodon T, Finke EW (2017). Makippugut (we are standing up): Public policy and self-determination in Nunavik. American Review of Canadian Studies.

[CR12] Fletcher, C., Lyonnais, M.-C., Saunders, I., Baron, A., Lynch, A., et al. (2021). *Definition of an Inuit cultural model and social determinants of health for Nunavik. Community Component.* (Nunavik Inuit Health Survey 2017 Qanuilirpitaa? How Are We Now?, p. 52).

[CR13] Fletcher, C., Riva, M., Lyonnais, M.-C., Baron, A., Saunders, I., Lynch, M., & Baron, M. (2022). Epistemic inclusion in the *Qanuilirpitaa?* Nunavik Inuit health survey: developing an Inuit model and determinants of health and well-being. 10.17269/s41997-022-00719-4 10.17269/s41997-022-00719-4 PMC10830955 36547790

[CR14] Gove, W. R., Hughes, M., & Galle, O. R. (1983). *Overcrowding in the household: An analysis of determinants and effects*. http://www.popline.org/node/400698

[CR15] Gray AP, Richer F, Harper S (2016). Individual-and community-level determinants of Inuit youth mental wellness. Canadian Journal of Public Health.

[CR16] Hamel, D., Hamel, G., & Gagnon, S. (2020). *Methodological report—QANUILIRPITAA? 2017 Nunavik Inuit Health Survey* (p. 429). Nunavik Regional Board of Health and Social Services. http://nrbhss.ca/sites/default/files/health_surveys/A11991_RESI_Rapport_methodologique_EP4.pdf

[CR17] Hansen, C. B., Larsen, C. V., Bjerregaard, P., & Riva, M. (2020). The effect of household crowding and composition on health in an Inuit cohort in Greenland. *Scandinavian Journal of Public Health, 1403494820929496*. 10.1177/1403494820929496 32508246

[CR18] Hervé C, Laneuville P (2017). La quête d’autonomie résidentielle des femmes inuites du Nunavik: Une perspective relationnelle. Recherches Amérindiennes Au Québec.

[CR19] Inuit Tapiriit Kanatami. (2016). *Inuit Tapiriit Kanatami’s 2016-2019 Strategy and Action Plan*. https://www.itk.ca/2016-2019-strategy-and-action-plan/

[CR20] Inuit Tapiriit Kanatami. (2019). *Inuit Nunangat housing strategy.* http://publications.gc.ca/collections/collection_2019/rcaanc-cirnac/R5-737-2019-eng.pdf

[CR21] Knotsch, C., & Kinnon, D. (2011). *If not now when? Addressing the ongoing Inuit housing crisis in Canada*. http://www.ruor.uottawa.ca/bitstream/10393/30246/1/2011_Inuit-Housing-Crisis-Canada-FullReport.pdf

[CR22] Kral MJ (2016). Suicide and suicide prevention among Inuit in Canada. The Canadian Journal of Psychiatry.

[CR23] Lachman ME, Weaver SL (1998). The sense of control as a moderator of social class differences in health and well-being. Journal of Personality and Social Psychology.

[CR24] Lauster N, Tester F (2010). Culture as a problem in linking material inequality to health: On residential crowding in the Arctic. Health & Place.

[CR25] Lepore SJ, Evans GW, Schneider ML (1991). Dynamic role of social support in the link between chronic stress and psychological distress. Journal of Personality and Social Psychology.

[CR26] Minich K, Saudny H, Lennie C, Wood M, Williamson-Bathory L, Cao Z, Egeland GM (2011). Inuit housing and homelessness: Results from the International Polar Year Inuit Health Survey 2007–2008. International Journal of Circumpolar Health.

[CR27] Muckle, G., Fletcher, C., Riva, M., Desrochers-Couture, M., Pepin, C., Poliakova, N., Moisan, C., Bélanger, R. E., & Fraser, S. (2020a). *Sociocultural determinants of health and wellness—QANUILIRPITAA? 2017 Nunavik Inuit Health Survey* (p. 89). Nunavik Regional Board of Health and Social Services.

[CR28] Muckle, G., Fraser, S., Desrochers-Couture, M., Pepin, C., Bélanger, R. E., Fletcher, C., Poliakova, N., & Moisan, C. (2020b). *Mental health and wellness—QANUILIRPITAA? 2017Nunavik Inuit Health Survey* (p. 45). Nunavik Regional Board of Health and Social Servic. http://nrbhss.ca/sites/default/files/health_surveys/A12528_RESI_Mental_Health_and_Wellness_EP5.pdf

[CR29] Pepin C, Muckle G, Moisan C, Forget-Dubois N, Riva M (2018). Household overcrowding and psychological distress among Nunavik Inuit adolescents: A longitudinal study. International Journal of Circumpolar Health.

[CR30] Perreault K, Riva M, Dufresne P, Fletcher C (2020). Overcrowding and sense of home in the Canadian Arctic. Housing Studies.

[CR31] Perreault, K., Dufresne, P., Potvin, L., & Riva, M. (2022a). *Housing as a determinant of Inuit mental health: Associations between improved housing measures and decline in psychological distress after rehousing in Nunavut and Nunavik, Canadian Journal of Public Health*. 10.17269/s41997-022-00701-0. 10.17269/s41997-022-00701-0 PMC10036679 36214994

[CR32] Perreault, K., Lapalme, J., Potvin, L., & Riva, M. (2022b). “We’re home now”: How a rehousing intervention shapes the mental well-being of Inuit adults in Nunavut, Canada. *International Journal of Environmental Research and Public Health, 19*(11). 10.3390/ijerph19116432 10.3390/ijerph19116432 PMC9180588 35682015

[CR33] Richmond CA (2009). The social determinants of Inuit health: A focus on social support in the Canadian Arctic. International Journal of Circumpolar Health.

[CR34] Riva M, Larsen CVL, Bjerregaard P (2014). Household crowding and psychosocial health among Inuit in Greenland. International Journal of Public Health.

[CR35] Riva, M., Plusquellec, P., Juster, R.-P., Laouan-Sidi, E. A., Abdous, B., Lucas, M., Dery, S., & Dewailly, E. (2014b). Household crowding is associated with higher allostatic load among the Inuit. *Journal of Epidemiology and Community Health*, jech-2013. 10.1136/jech-2013-203270 24385548

[CR36] Riva, M., Perreault, K., Dufresne, P., Fletcher, C., Muckle, G., Potvin, L., Bailie, R., & Baron, M. (2020). Social housing construction and improvements in housing outcomes for Inuit in Northern Canada. *Housing Studies*, 1–21. 10.17269/s41997-019-00249-6 PMC7046900 31741307

[CR37] Riva, M., Fletcher, C., Dufresne, P., Lachance, A., & Muckle, G. (2020a). *Housing and drinking water—QANUILIRPITAA? 2017 Nunavik Inuit Health Survey*. Nunavik Regional Board of Health and Social Services.

[CR38] Riva, M., Fletcher, C., Dufresne, P., Lachance, A., & Muckle, G. (2020b). *Sociodemographic characteristics—QANUILIRPITAA? 2017 Nunavik Inuit Health Survey*. Nunavik Regional Board of Health and Social Services.

[CR39] Sigouin C, Charpentier M, Quéniart A (2010). La grand-maternité chez les Inuits: Portrait d’une réalité méconnue. Nouvelles Pratiques Sociales.

[CR40] Société d’habitation du Québec. (2014). *Housing in Nunavik*. http://www.habitation.gouv.qc.ca/fileadmin/internet/documents/English/logement__nunavik_2014.pdf

[CR41] Standing Senate Committee on Aboriginal Peoples. (2017). *We Can Do Better*. https://sencanada.ca/content/sen/committee/421/APPA/Reports/Housing_e.pdf

[CR42] Statistics Canada. (2001). *2001 Aboriginal Peoples Survey Questionnaire*. file:///C:/Users/charl/AppData/Local/Temp/3250_Q1_V1-eng.pdf

[CR43] Statistics Canada. (2015). *Projections de la population et des ménages autochtones au Canada, 2011 à 2036*.

[CR44] Statistics Canada. (2017). *Census in brief: The housing conditions of Aboriginal peoples in Canada, 2016.* https://www12.statcan.gc.ca/census-recensement/2016/as-sa/98-200-x/2016021/98-200-×2016021-eng.cfm

[CR45] Stern P (2005). Wage labor, housing policy, and the nucleation of Inuit households. Arctic Anthropology.

[CR46] Therrien A, Duhaime G (2017). Le logement social au Nunavik: Pouvoirs et responsabilités. Recherches Amérindiennes Au Québec.

[CR47] Wells NM, Harris JD (2007). Housing quality, psychological distress, and the mediating role of social withdrawal: A longitudinal study of low-income women. Journal of Environmental Psychology.

[CR48] Wenzel GW (2000). Sharing, money and modern Inuit subsistence: Obligation and reciprocity at Clyde River, Nunavut. Senri Ethnological Studies.

